# Biocatalytic Detoxification of Ochratoxins A/B by a Fungal Dye-Decolorizing Peroxidase: Mechanistic Insights and Toxicity Assessment

**DOI:** 10.3390/toxins17090438

**Published:** 2025-09-02

**Authors:** Wenjing Xia, Nianqing Zhu, Jie Mei, Yueqin Peng, Fanglin Song, Shuai Ding, Fei Li, Xue Zhou

**Affiliations:** 1 College of Chemical and Biological Engineering, Nanjing Normal University Taizhou College, Taizhou 225300, China; xiawenjing1234@163.com (W.X.);; 2 Jiangsu Key Laboratory of Chiral Pharmaceuticals Biosynthesis, College of Pharmacy and Chemistry & Chemical Engineering, Taizhou University, Taizhou 225300, China; 3 Department of Bioengineering, School of Chemistry and Chemical Engineering, Wuhan University of Science and Technology, Wuhan 430081, China

**Keywords:** ochratoxin A, ochratoxin B, dye-decolorizing peroxidase, detoxification

## Abstract

Mycotoxin contamination in agricultural products poses severe global health risks, with ochratoxins (particularly OTA and OTB) exhibiting marked nephrotoxicity and classified as Group 2B carcinogens by IARC. Conventional physical/chemical detoxification methods often impair food nutritional quality, highlighting the need for enzymatic alternatives. Herein, we systematically investigated the degradation mechanisms of ochratoxin A (OTA) and ochratoxin B (OTB) using *Pleurotus ostreatus* dye-decolorizing peroxidase (*Po*DyP4) coupled with redox mediators. Remarkably, hydroxybenzotriazole (HBT) enhanced degradation efficiency 26.7-fold for OTA and 10.6-fold for OTB compared to mediator-free systems, establishing it as the optimal catalytic enhancer. Through LC-MS/MS analysis, we identified five key degradation products, including 6-OH-OTA and OTB-quinone, elucidating a putative oxidative degradation pathway. In vitro cytotoxicological evaluation in HK-2 cells demonstrated that *Po*DyP4-treated ochratoxins significantly attenuated cytotoxicity, reducing malondialdehyde (MDA) levels by 48.7% (OTA) and 42.3% (OTB) (*p* < 0.01) and suppressing ROS generation. Molecular docking revealed strong binding affinities between *Po*DyP4 and ochratoxins, with calculated binding energies of −7.6 kcal/mol (OTA) and −8.6 kcal/mol (OTB), stabilized by hydrogen bond networks (1.9–3.4 Å). These findings position *Po*DyP4 as a promising biocatalyst for mycotoxin mitigation in food systems, offering a sustainable alternative to traditional detoxification methods.

## 1. Introduction

Mycotoxins, as toxic secondary metabolites produced by filamentous fungi, pose severe threats to global food safety. The contamination of agricultural commodities with these toxic compounds not only jeopardizes human and animal health but also causes substantial economic losses throughout the food supply chain. According to Food and Agriculture Organization (FAO) estimates, over 25% of global food crops are contaminated with mycotoxins annually [1]. Among the more than 400 identified mycotoxins, ochratoxin is one of the most prevalent regulated contaminants in the food system [2,3].

The ochratoxin family, predominantly produced by Aspergillus and Penicillium species [4], comprises more than 20 structural analogs. These include ochratoxin A (OTA), ochratoxin B (OTB), and ochratoxin C (OTC), along with their non-amide derivatives (OTα, OTβ), and hydroxylated forms (4R-OH-OTA, 4S-OH-OTA, etc.) [5,6]. Of particular concern is OTA, classified as a Group 2B human carcinogen by the International Agency for Research on Cancer (IARC), which exhibits multisystem toxicity including nephrotoxic, hepatotoxic, and genotoxic effects [7,8]. In China, regulatory limits for OTA are strictly enforced at 5 μg/kg in foods [9] and 100 μg/kg in feeds [10]. Although OTB demonstrates lower toxicity than its chlorinated counterpart OTA [11],its co-occurrence with OTA and potential bioconversion warrant equal attention [12].

Mycotoxin biosynthesis is highly dependent on environmental conditions, including temperature, water activity, pH, and microbial interactions [13]. While proper grain storage can mitigate fungal growth [14], complete prevention remains challenging due to contamination risks across pre-harvest, harvest, and post-harvest stages [15]. Current detoxification strategies fall into three categories: physical methods (e.g., adsorption, irradiation), chemical treatments (e.g., ozonation, alkalization), and biological approaches (microbial/enzymatic degradation) [16,17,18]. Among these, biological detoxification has emerged as the most promising solution due to its specificity, environmental compatibility, and preservation of food quality [19,20]. Microbial degradation of OTA primarily occurs through enzymatic pathways, with the first OTA-degrading enzyme reported in 1969 [21]. Recent advances have identified multiple enzymatic mechanisms including amide bond hydrolysis (yielding OTα + L-β-phenylalanine), isocoumarin ring hydroxylation/dechlorination, and lactone ring opening [22,23].

Dye-decolorizing peroxidases (DyPs; EC 1.11.1.19) represent a novel biocatalytic tool for mycotoxin remediation. These heme-containing oxidoreductases utilize H_2_O_2_ to degrade recalcitrant compounds through high-redox-potential reactions [24]. While DyPs have shown efficacy against ZEN [25] and aflatoxin B_1_ [26], their potential for ochratoxin degradation remains underexplored.

This study therefore investigates the degradation kinetics and detoxification mechanisms of OTA/OTB by DyP4 from *Pleurotus ostreatus* (*Po*DyP4). In addition, the cytotoxicity of the degradation product was further evaluated and molecular interactions through docking simulations were also further revealed.

## 2. Results and Discussion

### 2.1. Degradation Properties of Ochratoxin A and B with PoDyP4

**Figure 1:**
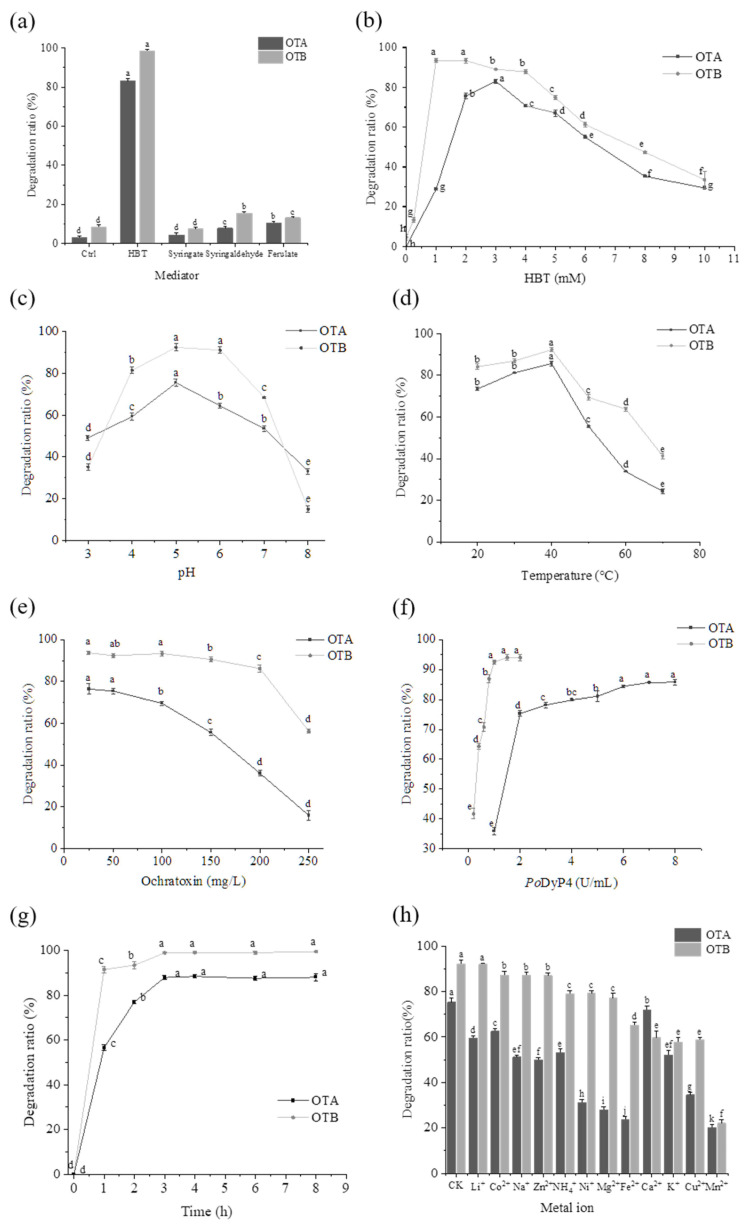
The effects of (**a**) mediator, (**b**) mediator concentration, (**c**) pH, (**d**) temperature, (**e**) ochratoxin concentration, (**f**) enzyme concentration, (**g**) reaction time, and (**h**) ion type on the degradation of OTA and OTB by *Po*DyP4. Different letters indicate significant differences (*p* < 0.05), whereas the same letters indicate no significant differences.

Under controlled experimental conditions, the dye-decolorizing peroxidase *Po*DyP4 demonstrated robust degradation activity against both ochratoxin A (OTA) and ochratoxin B (OTB). Comprehensive evaluation of reaction parameters revealed that hydroxybenzotriazole (HBT) served as the most effective redox mediator, enabling degradation efficiencies of 83.04% for OTA and 98.34% for OTB, significantly outperforming alternative mediators including syringate (4.42% OTA, 7.41% OTB), syringaldehyde (7.73% OTA, 15.48% OTB), and ferulate (10.34% OTA, 13.12% OTB) (*p* < 0.01) (Figure 1a). The influence of varying HBT concentrations on degradation rates was also investigated (Figure 1b), indicating that HBT concentrations of 3 mM and 1 mM optimize the degradation of OTA and OTB most effectively.

The relationship between pH and degradation rate demonstrates a skewed bell-shaped curve characterized by a subtle acidic preference (Figure 1c), while the optimal pH for *Po*DyP4 to effectively degrade OTA and OTB in the presence of the mediator HBT was 5.0. As the pH level neared 8.0, there was a significant reduction in the degradation rate, dropping to 33.03% for OTA and 14.53% for OTB, which suggests that *Po*DyP4 is not appropriate for mildly alkaline conditions. Nobre et al. [27] previously investigated the degradation characteristics of OTA and ZEN using *Pleurotus ostreatus* powder, demonstrating comparable findings.

The influence of varying temperatures on the degradation of ochratoxin by *Po*DyP4 is depicted in Figure 1d. As the temperature increases, the degradation ratio shows a gradual rise from 20 °C to 40 °C, followed by a significant decline from 40 °C to 70 °C for both OTA and OTB, suggesting that *Po*DyP4 exhibits enhanced degradation efficiency for OTA under moderate and low temperature conditions.

Other individual factors, such as reaction time, substrate concentration, and enzyme concentration, were also examined and are presented in Figure 1e–g. Specifically, at a substrate level of 50 mg/L, the critical concentration and critical reaction duration for *Po*DyP4 were found to be 2 U/mL and 3 h, respectively. An elevation in ochratoxin concentration negatively influenced the degradation performance of *Po*DyP4; when the OTB level was increased from 25 to 200, there was only a 7.54% reduction in the degradation rate, while an increase in OTA concentration led to a substantial decrease of 40.24%.

Different metal ions exhibit distinct levels of inhibition regarding the degradation rates of OTA and OTB (Figure 1h). Specifically, the inhibitory effect on OTA is more significant than that on OTB. Among these ions, Ca^2+^ has the least impact on OTA, whereas Li^+^ shows a relatively smaller effect on OTB. Mn^2+^ exerts the greatest inhibitory influence on both types of ochratoxins.

These comprehensive parameter optimizations establish that *Po*DyP4 as an effective biocatalyst for ochratoxin degradation under carefully controlled conditions.

### 2.2. Degradation Products of Ochratoxin A and B and Their Pathways

**Figure 2:**
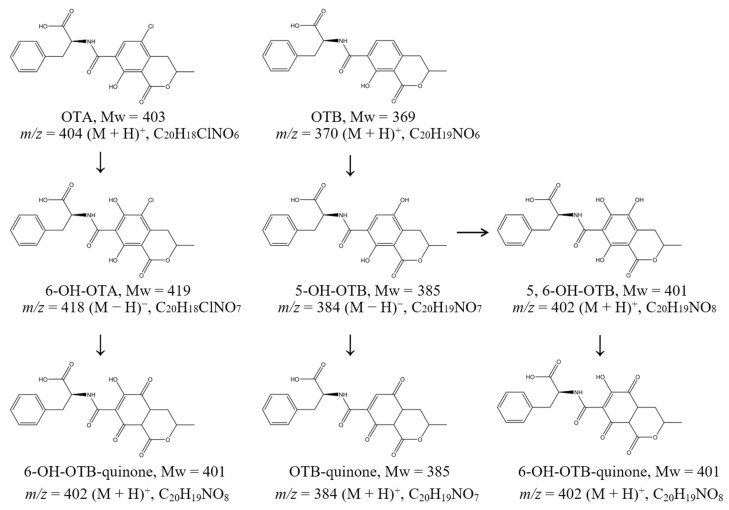
The reaction pathways of *Po*DyP4 in degrading OTA and OTB.

Through comprehensive UPLC-MS analysis, we identified five principal degradation products and elucidated the metabolic pathways of OTA and OTB mediated by *Po*DyP4 (Figure 2). The mass spectra revealed characteristic molecular ion peaks at *m*/*z* 404 [M + H]^+^ for OTA and *m*/*z* 370 [M + H]^+^ for OTB, with corresponding adduct ions observed at *m*/*z* 426 [M + Na]^+^ and *m*/*z* 442 [M + K]^+^ for OTA, and *m*/*z* 392 [M + Na]^+^ and *m*/*z* 408 [M + K]^+^ for OTB (Supplementary Figure S2a,b).

The degradation pathway initiates with regioselective hydroxylation at the C6 position of OTA, generating 6-hydroxy-OTA (C_20_H_18_ClNO_7_; *m*/*z* 420 [M + H]^+^), which subsequently undergoes oxidative transformation to form 6-hydroxy-OTB quinone (C_20_H_19_NO_8_; *m*/*z* 402 [M + H]^+^ and *m*/*z* 400 [M − H]^−^). Parallel analysis of OTB metabolism demonstrated analogous hydroxylation events, with initial formation of 5-hydroxy-OTB (C_20_H_19_NO_7_; *m*/*z* 384 [M − H]^−^) that was either directly oxidized to OTB quinone (C_20_H_19_NO_7_; *m*/*z* 384 [M − H]^−^) or further hydroxylated to yield 5,6-dihydroxy-OTB (C_20_H_19_NO_8_; *m*/*z* 402 [M + H]^+^). The latter intermediate was ultimately converted to 6-hydroxy-OTB quinone (C_20_H_19_NO_8_; *m*/*z* 402 [M + H]^+^), completing the metabolic cascade (Supplementary Figure S2c–g). This degradation pathway markedly differs from those of esterases, lipases, and other hydrolase classes [28,29], as it does not yield OTα or OTβ. A systematic cytotoxicity assessment will subsequently be conducted on the resultant products.

### 2.3. The Assessment of Cytotoxicity of Degradation Products on HK-2 Cells

**Figure 3:**
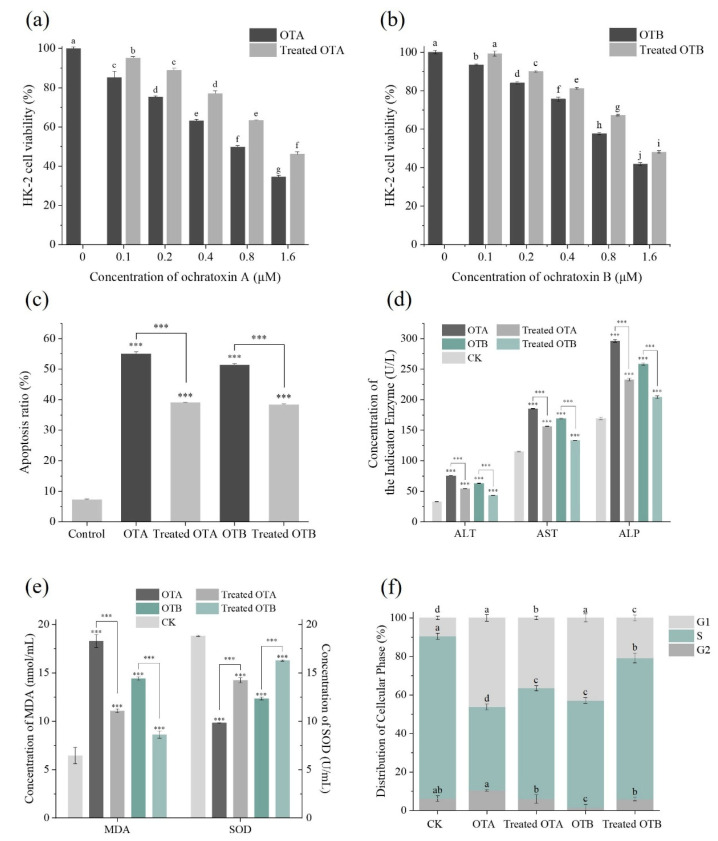
(**a**) The survival rate of cells exposed to different concentrations of degraded and undegraded OTA. (**b**) The survival rate of cells exposed to different concentrations of degraded and undegraded OTB. (**c**) The apoptosis rate of cells in OTA and OTB before (0.8 µM) and after degradation. (**d**) The concentrations of extracellular ALT, AST and ALP before and after degradation treated with OTA and OTB. (**e**) The concentrations of MDA and SOD in cells before and after degradation treated with OTA and OTB. (**f**) Cell cycle alterations in response to OTA and OTB treatment before and after degradation. Different letters indicate significant differences (*p* < 0.05), whereas the same letters indicate no significant differences. Additionally, *** denotes statistical significance at the *p* < 0.001 level.

Prior investigations have systematically evaluated the cytotoxicological profiles of OTA and OTB through both in vitro and in vivo approaches [30,31]. The cytotoxicity assessment revealed a marked concentration-dependent reduction in HK-2 cell viability following exposure to both OTA and OTB compared to blank controls (Figure 3a,b). Notably, enzymatic treatment with *Po*DyP4 substantially attenuated these toxic effects, reducing cytotoxicity to levels comparable to half the original toxin concentrations. This protective effect was further evidenced by significant inhibition of ochratoxin-induced apoptosis, with apoptotic rates declining from 55.01% to 39.01% for OTA and from 51.24% to 38.36% for OTB following *Po*DyP4 treatment (Figure 3c).

Analysis of hepatic biomarkers demonstrated pronounced elevation of ALT, AST, and ALP levels in OTA- and OTB-treated groups relative to controls, indicating cellular damage. However, *Po*DyP4 intervention effectively normalized these enzyme concentrations (Figure 3d), suggesting restoration of cellular integrity. Concurrent evaluation of oxidative stress markers revealed that ochratoxin exposure significantly increased malondialdehyde (MDA) levels while suppressing superoxide dismutase (SOD) activity [32,33], both of which were significantly ameliorated by *Po*DyP4 treatment (Figure 3e).

**Figure 4:**
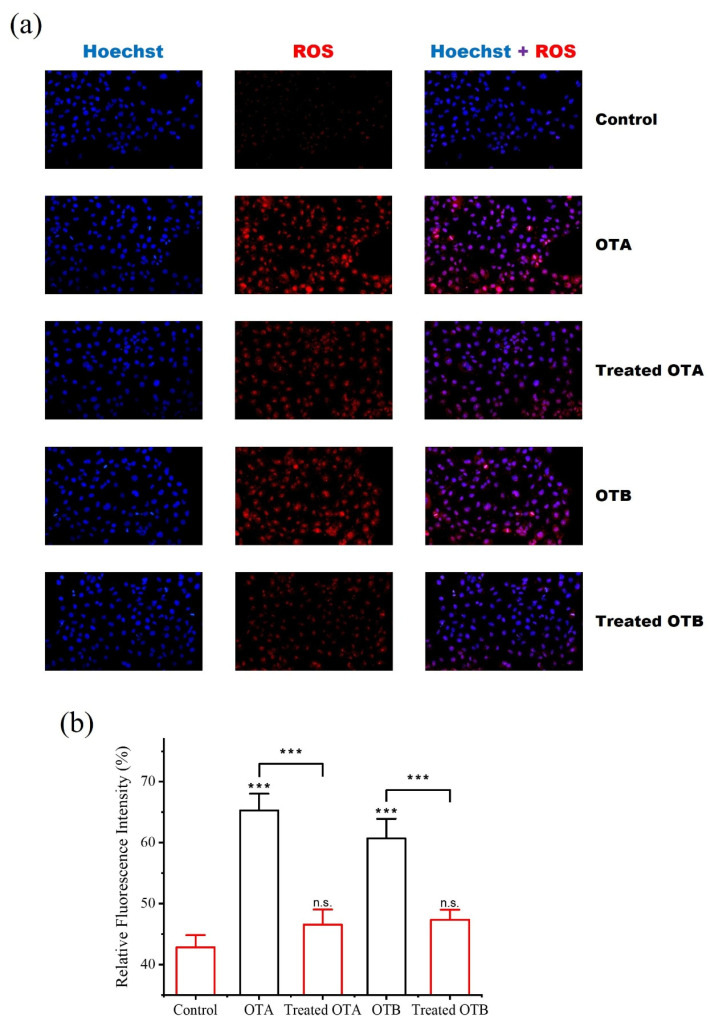
(**a**) Determination of ROS level by fluorescence. (**b**) Relative fluorescence intensity. n.s. indicates no significant difference, and *** indicates significance at *p* < 0.001.

Cell cycle analysis showed that OTA and OTB exposure caused G1 phase arrest in HK-2 cells, consistent with ROS-mediated growth inhibition [34]. Following *Po*DyP4 treatment, we observed a significant increase in S-phase cell populations (Figure 3f), indicating restored proliferative capacity. These findings were corroborated by fluorescence staining assays, which confirmed substantial reduction of intracellular ROS levels after enzymatic detoxification (Figure 4a,b).

### 2.4. Molecular Binding Mode of PoDyP4 with Ochratoxin A and B

**Figure 5:**
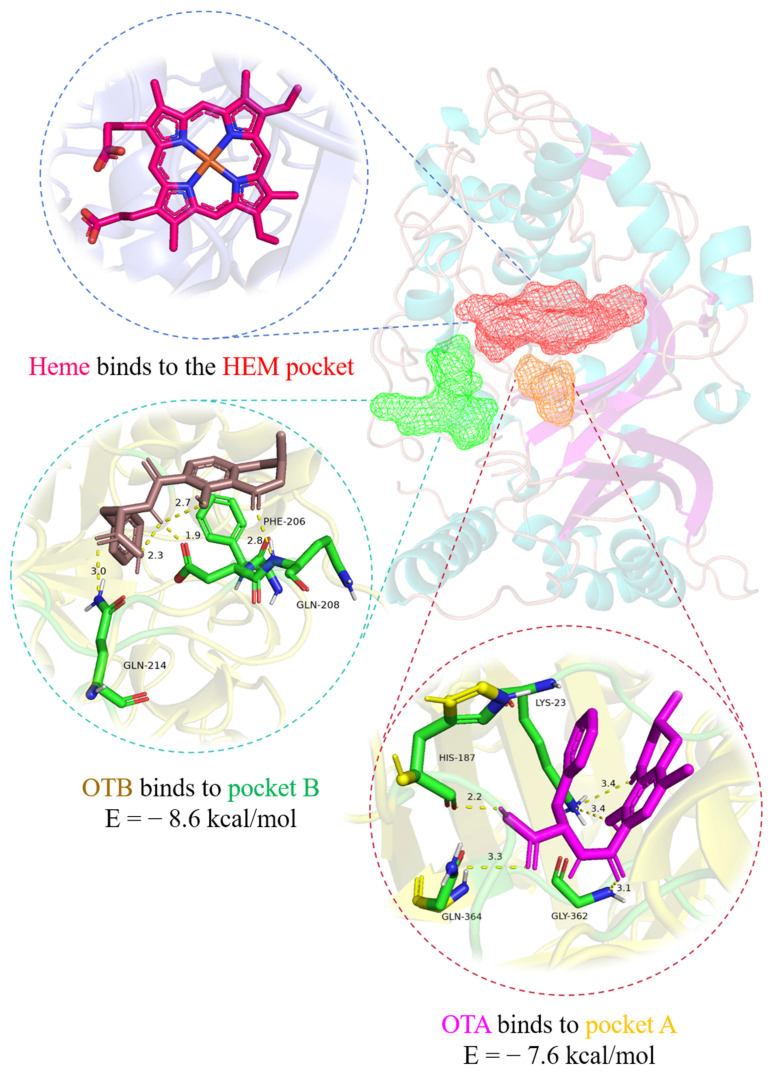
The docking results of *Po*DyP4 with OTA and OTB.

Structural analysis of *Po*DyP4 revealed three distinct binding pockets: the heme-binding pocket (HEM pocket), along with two adjacent substrate-binding pockets designated as Pocket A and Pocket B (Figure 5). While the HEM pocket accommodates the natural heme cofactor, molecular docking studies demonstrated that Pocket A and Pocket B preferentially bind ochratoxin A (OTA) and ochratoxin B (OTB), respectively. Detailed examination of the binding interfaces identified key stabilizing interactions, with OTA forming five hydrogen bonds involving residues Lys-23, His-187, Gly-362, and Gln-364, while OTB established three hydrogen bonds with Phe-206, Gln-208, and Gln-214. Although molecular docking cannot fully capture the dynamic catalytic process, the structural configuration of these expanded substrate-binding pockets adjacent to the heme center provides compelling evidence for *Po*DyP4’s promiscuous substrate specificity, suggesting its potential as a versatile biocatalyst for diverse toxin degradation.

## 3. Conclusions

This comprehensive study demonstrates the remarkable efficacy of *Po*DyP4 in degrading ochratoxin A and B through systematic evaluation of various redox mediator systems. Our findings establish hydroxybenzotriazole (HBT) as the optimal mediator, significantly enhancing degradation efficiency compared to mediator-free systems. Through rigorous parameter optimization, we identified the ideal reaction conditions, encompassing temperature, pH, incubation time, and metal ion tolerance, for maximal enzymatic activity. Structural characterization via UPLC-MS/MS revealed five primary degradation products, enabling elucidation of the complete metabolic pathway. Importantly, cell-based assays confirmed the substantial reduction in ochratoxin-induced cytotoxicity following *Po*DyP4 treatment, as evidenced by normalized biomarkers of cellular damage and oxidative stress. Molecular docking analyses provided structural insights into the enzyme-substrate interactions, with favorable binding energies and specific hydrogen bonding patterns explaining the observed catalytic efficiency. These collective findings position *Po*DyP4 as a highly promising biocatalyst for food safety applications, offering an effective enzymatic strategy for ochratoxin mitigation while preserving food quality.

## 4. Materials and Methods

### 4.1. Substrates and Chemicals

Ochratoxin A and B standards (abbreviated as OTA and OTB) were provided by Aladdin (Shanghai, China). The enzyme of *Po*DyP4 used in this study was kindly provided by the Prof. Fei Li’s group from Wuhan University of Science and Technology [25]. Human renal tubular epithelial cells (HK-2) were provided by Wuhan Baiqandu Biotechnology (Wuhan, China). The kits used for analyzing the biochemical indicators of HK-2 cells in the presence of OTA/OTB and their metabolites including the malondialdehyde (MDA) test kit and the superoxide dismutase (SOD) determination kit were provided by Jianchen Biotechnology (Nanjing, China); the aspartate aminotransferase (AST) kit, alanine aminotransferase (ALT) kit and alkaline phosphatase (ALP) kit were provided by BIOBASE (Bornheim, Germany). A Cell Counting Kit-8 (CCK8) was purchased to analyze the vitality of the cells from Meilunbio (Dalian, China). Fetal Bovine Serum (FBS) and α-MEM liquid medium for cells growth were purchased from HAKATA (Shanghai, China) and Biosharp (Wuhan, China), respectively. Annexin V-FITC/PI double staining apoptosis detection kits were acquired from Bestbio Biotechnology (Shanghai, China). An ROS Assay Kit and fluorescent dye were provided by Beyotime (Beijing, China). All other reagents including 2,2′-azino-bis-(3-ethylbenzothiazoline-6-sulphonic acid) (ABTS), 1-hydroxybenzotriazole (HBT), syringate, syringaldehyde, and ferulate were of analytical grade or higher and acquired from Sinopharm Chemical Reagent (Beijing, China).

### 4.2. Enzymatic Degradation of Ochratoxin A and B by PoDyP4

The enzyme activity of recombinant *Po*DyP4 was assayed spectrophotometrically using ABTS (ε_420nm_ = 36,000 M^−1^ cm^−1^) as the substrate. The standard test conditions are as follows: 40 mM Britton–Robinson (BR) buffer (pH 4.0), 0.3 mM ABTS, a specified volume of enzyme solution, and 0.25 mM H_2_O_2_ at a temperature of 30 °C. One unit of DyP activity was defined as the amount of enzyme required to oxidize 1 µmol ABTS per minute.

The influence of various individual factors on degradation rates was systematically investigated, including pH (3, 4, 5, 6, 7, and 8), temperature (20 °C, 30 °C, 40 °C, 50 °C, 60 °C, and 70 °C), reaction duration (1, 2, 3, 4, 6, and 8 h), mediator (HBT, syringate, syringaldehyde, and ferulate, with 5 mM for 18 h), concentrations of *Po*DyP4 for OTA degradation (ranging from 1 to 8 U/mL) and OTB degradation (from 0.2 to 2 U/mL), as well as the initial concentrations of OTA and OTB (25 to 250 mg/L). Additionally, variations in the mediator HBT concentration were considered (0 to 10 mM) along with the effects of metal ions such as Cu^2+^, Na^+^, Co^2+^, Mn^2+^, K^+^, Li^+^, Ni^2+^, Ca^2+^, Mg^2+^, Fe^2+^, and Zn^2+^ and non-metal ion NH_4_^+^ on the overall degradation rate. Unless otherwise specified, the degradation reactions were conducted in a total volume of 100 µL at 30 °C for 2 h containing 50 mg/L of ochratoxin, 2 U/mL (OTA) or 1 U/mL (OTB) of *Po*DyP4, 5 mM of H_2_O_2_ and 40 mM BR Buffer (pH 5.0). A control group was established without the inclusion of *Po*DyP4. All experimental treatments were replicated three times.

The peak patterns of OTA and OTB at various ultraviolet wavelengths (Supplementary Figure S1) were analyzed using an HPLC system (Agilent 1220) equipped with a C18 reverse phase column (Agilent, 5 μm, 4.6 mm × 250 mm) in order to determine the optimal spectroscopic conditions.

Subsequent to the addition of an equivalent volume of acetonitrile to halt the degradation process of OTA and OTB, the samples were subjected to filtration through a 0.22 μm organic filter membrane. A 20 μL sample was injected and analyzed with phase A-B (37:63, *v*/*v*; phase A: acetonitrile, phase B: water-acetonitrile-CH_3_COOH in a volume ratio of 89:10:1) as the mobile phase at a flow rate of 2 mL/min and a temperature of 30 °C, with detection wavelengths set at 330 nm for OTA and 318 nm for OTB. The degradation ratio of ochratoxin was calculated using the following formula: Degradation ratio (%) = (Cc − Cs)/Cc × 100, where Cc and Cs represent the concentrations of ochratoxin in the control and sample, respectively.

Statistical analyses were performed using IBM SPSS Statistics 27, and graphical representations were generated with OriginPro 2024.

### 4.3. Detection and Identification of Ochratoxin A and B and Their Degradation Products

For the identification of OTA and OTB degradation products, an ultra-high performance liquid chromatography system equipped with a Hypersil GOLD column (Thermo Scientific, 2.1 mm × 100 mm, 3 μm, Waltham, MA, USA) coupled with a mass spectrometry system (Thermo Scientific, Q Exactive, Waltham, MA, USA) was employed. The MS system operated with a spray voltage (ESI ion source) of 3200 V and a capillary temperature of 300 °C. Data acquisition was performed in both positive and negative ion modes.

### 4.4. HK-2 Cell Viability and Apoptosis Assay

The cytotoxic effects of ochratoxin A and B were evaluated using human kidney-2 (HK-2) cells. HK-2 cells in the logarithmic growth phase were treated with trypsin, and the resulting cell suspension was then plated into a 96-well plate at a density of 6000 cells per well. The plates were incubated for 24 h in DMEM culture medium supplemented with 10% FBS. Untreated ochratoxin and degradation products of ochratoxin generated by *Po*DyP4 were added separately at final concentrations of 0, 0.1, 0.2, 0.4, 0.8, 1.6, 3.2, 6.4, and 12.8 µM and co-cultured with pretreated HK-2 cells in a CO_2_ incubator for a duration of 48 h.

In the cell viability assay, each well received 100 µL of fresh 10% CCK-8 solution to substitute the culture medium. After an additional incubation for 2 h at 37 °C, absorbance was measured at a wavelength of 450 nm. The method for determining cell viability (%) is outlined as follows: (As − Ab)/(Ac − Ab) × 100%, where As indicates the absorbance of samples subjected to drug treatment, while Ac refers to the absorbance of the control group that did not receive any treatment.

In the cell apoptosis assay, logarithmic growth HK-2 cells were digested with trypsin, cultured in 6-well plates as before, and treated with 0.8 µM of ochratoxin and degradation products for 48 h, respectively. Following this, HK-2 cells were collected, rinsed with pre-cooled PBS buffer, and then resuspended in 5 µL Annexin V-FITC, 5 µL propidium iodide (PI), and 100 µL binding buffer for a 10-min incubation at room temperature in the dark. After adding an additional 400 µL of binding buffer, the samples were analyzed using flow cytometry with an excitation wavelength of 488 nm and an emission wavelength of 530 nm to detect the signal.

To evaluate the cell cycle distribution, intracellular DNA content was measured in HK-2 cells that underwent identical treatment to that described above. Following overnight fixation with 75% cold ethanol, HK-2 cells were incubated with a staining solution composed of RNase A and PI at a ratio of 1:9 and subsequently analyzed by flow cytometry at an excitation wavelength of 561 nm.

### 4.5. Biochemical Index Analysis of HK-2 Cells

To further elucidate the changes in toxicity before and after the degradation of OTA and OTB, liver function markers including alanine aminotransferase (ALT), aspartate aminotransferase (AST), and alkaline phosphatase (ALP), as well as reactive oxygen species (ROS) levels, such as superoxide dismutase (SOD) and malondialdehyde (MDA) were measured with automatic biochemical analyzer (BIOBASE Co., Ltd., Jinan, China) and microplate reader (USCN Business Co., Ltd., Wuhan, China). Additionally, fluorescence ROS staining experiments were performed on cells labeled with 10 µmol/L superoxide anion probes (Ex = 300 nm, Em = 610 nm; ROS Assay Kit for Superoxide Anion with DHE, Beyotime, Beijing, China), using 5 µg/mL Hoechst 33342 (Ex = 350 nm, Em = 461 nm; Beyotime, Beijing, China) staining as a reference. Images were collected using an inverted fluorescence microscope and quantified with the ImageJ 1.54. The detailed analysis methods of MDA, SOD, ALT, AST, ALP, and ROS can be found in the Supplementary Materials.

### 4.6. Molecular Docking of PoDyP4 with Ochratoxin A and B

To elucidate the binding mechanism of OTA and OTB to *Po*DyP4, molecular docking simulations were conducted utilizing AutoDock Vina 1.1.2 [35]. The structural model of *Po*DyP4 was constructed based on the SWISS-MODEL platform (https://swissmodel.expasy.org/) using the 3D structure of DyP (PDB entry: 6FSK) as a template. Subsequently, the protein binding sites were predicted using POCASA 1.1. The structures of *Po*DyP4, OTA, and OTB were processed for hydrogen addition, charge calculation, and assignment using AutodockTools 1.5.6. Visualization and analysis of the interaction patterns from the docking results were performed using PyMOL 2.3.0.

## Data Availability

The original contributions presented in this study are included in the article and Supplementary Materials. Further inquiries can be directed to the corresponding authors.

## Supplementary Materials

The following supporting information can be downloaded at: https://www.mdpi.com/article/10.3390/toxins17090438/s1. Figure S1. HPLC analysis of OTA of OTB; Figure S2. LC-MS analysis of OTA, OTB and their degradation products.
