# Supplementary material for: Biocatalytic Detoxification of Ochratoxins A/B by a Fungal Dye-Decolorizing Peroxidase: Mechanistic Insights and Toxicity Assessment
Source: Toxins (Basel). 2025 Sep 2;17(9):438. doi: 10.3390/toxins17090438 (PMC12474158; doi:10.3390/toxins17090438)
Supplement: Supplementary file 1 [file toxins-17-00438-s001.zip › toxins-3779331-supplementary.pdf]

Figure S1. HPLC analysis of OTA of OTB

(a)

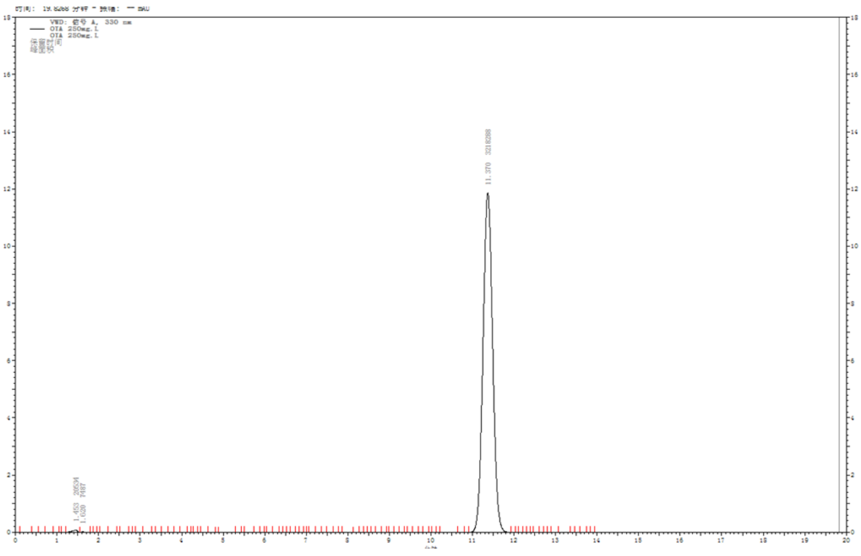

HPLC analysis of OTA

(b)

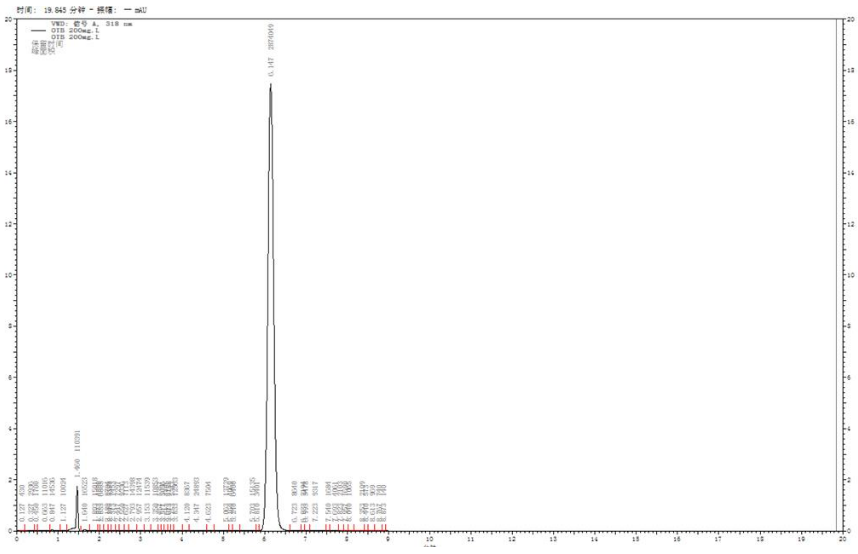

HPLC analysis of OTB

Figure S2. LC-MS analysis of OTA, OTB and their degradation products

(a) OTA

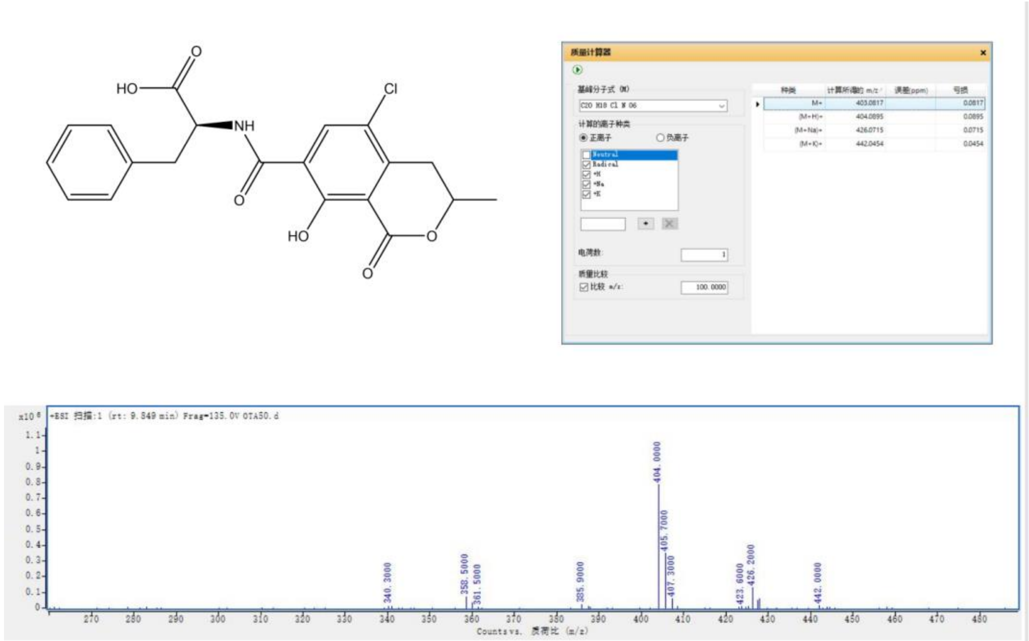

(b) OTB

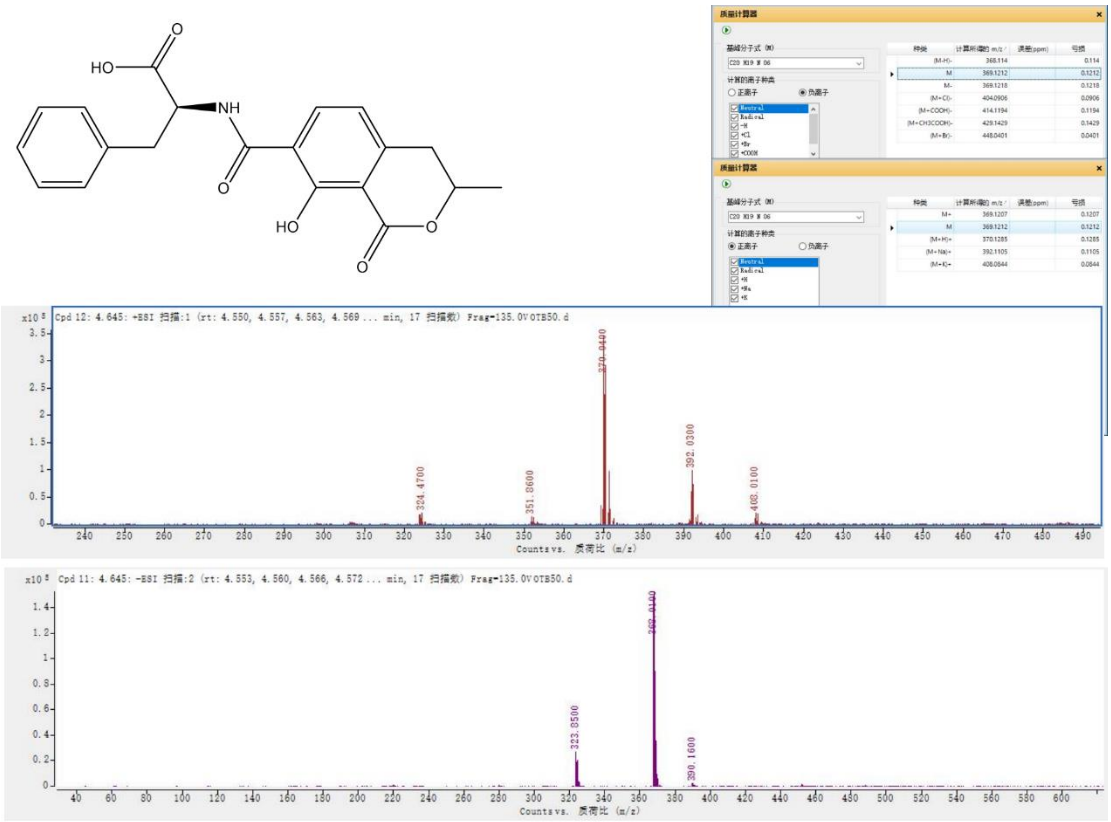

(c) 6-OH-OTA

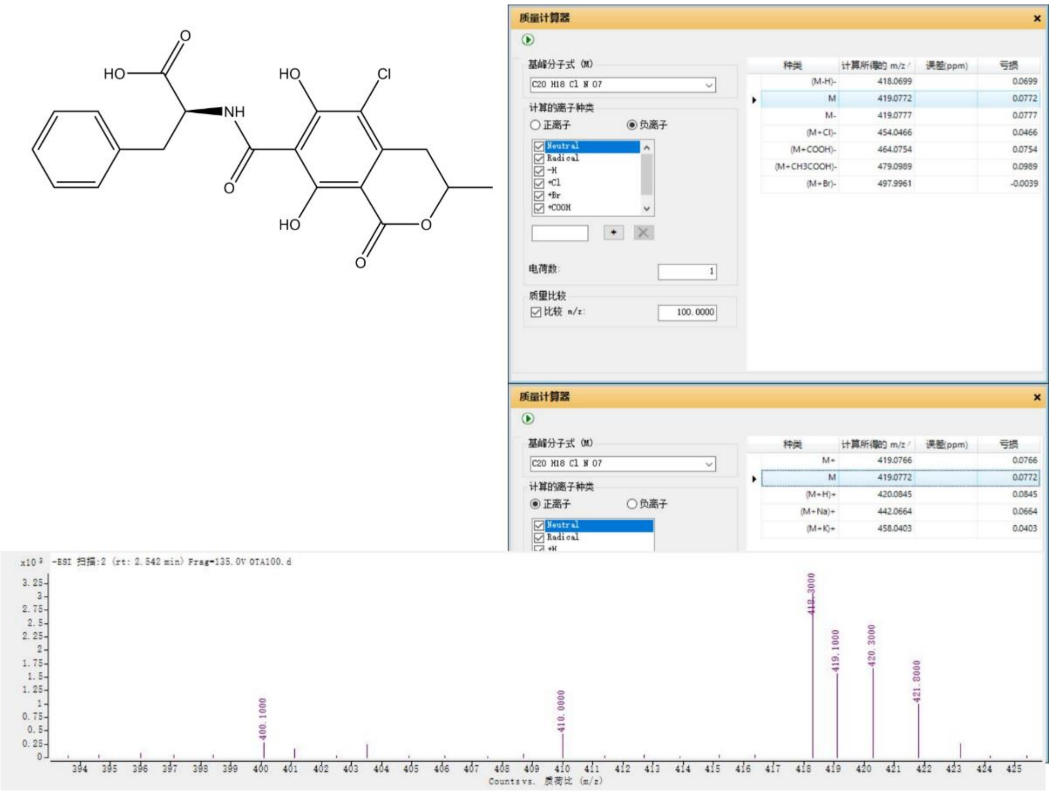

(d) 6-OH-OTB-quinone(from OTA)

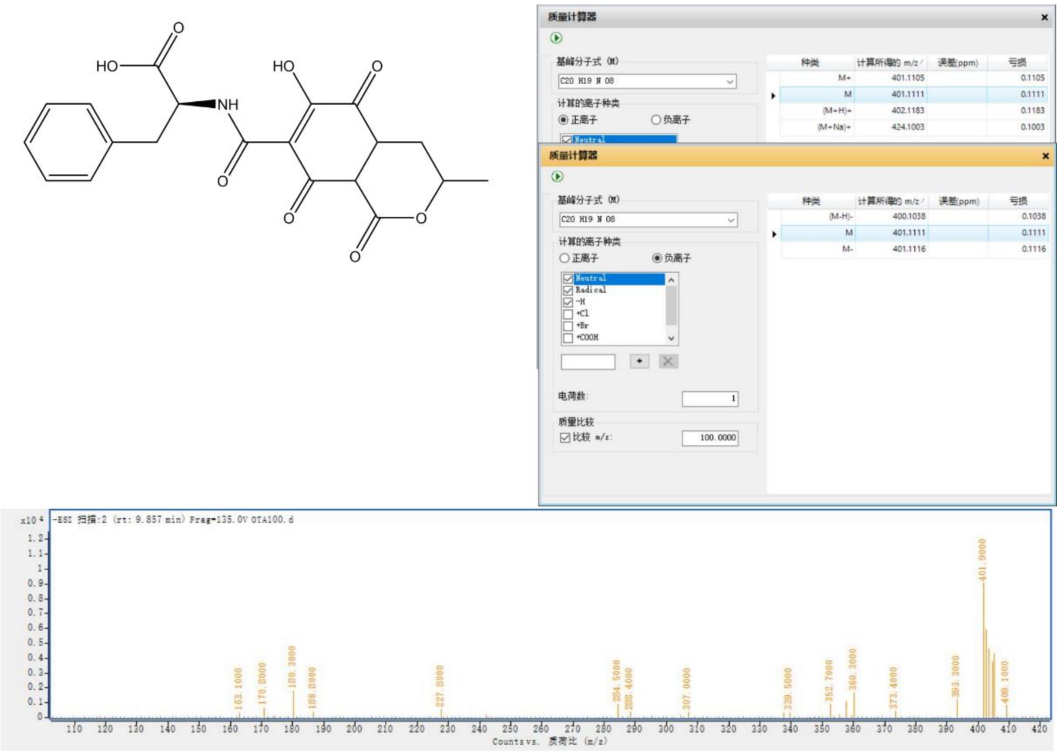

(e) 5-OH-OTB

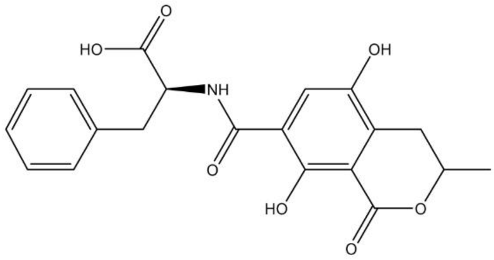

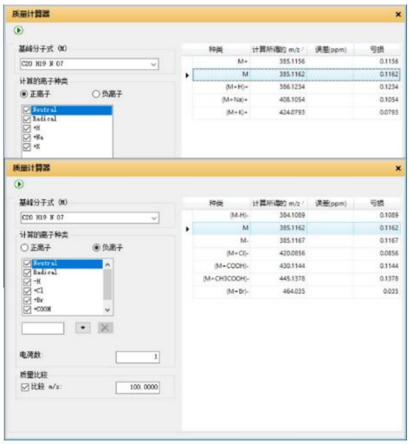

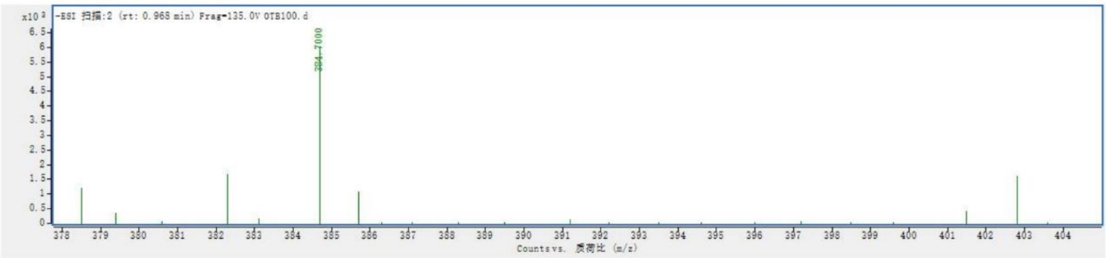

(f) OTB-quinone

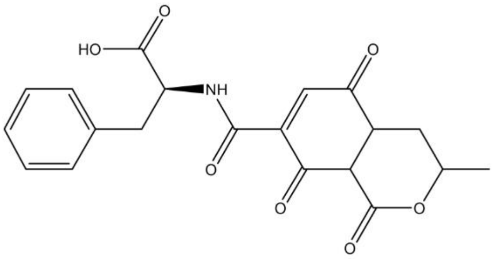

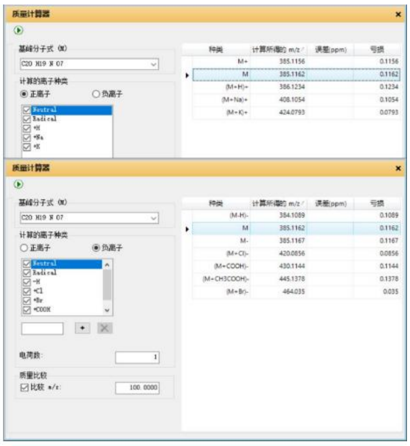

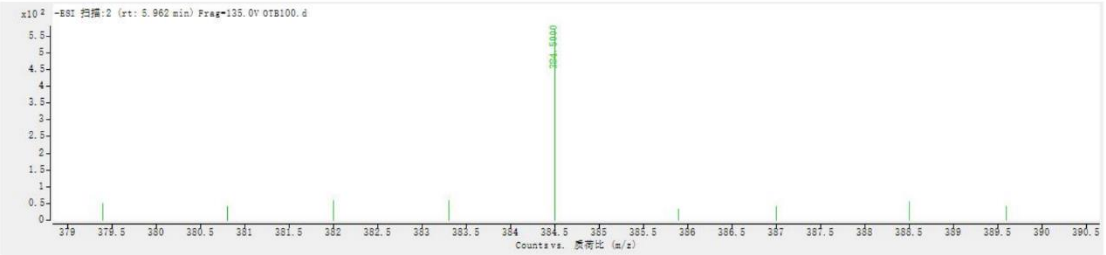

(g) 5,6-OH-OTB

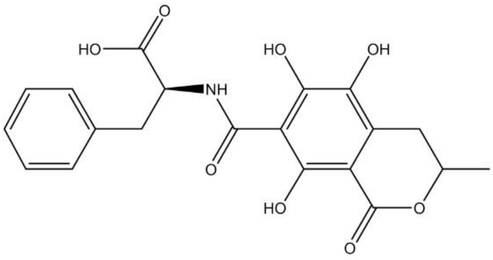

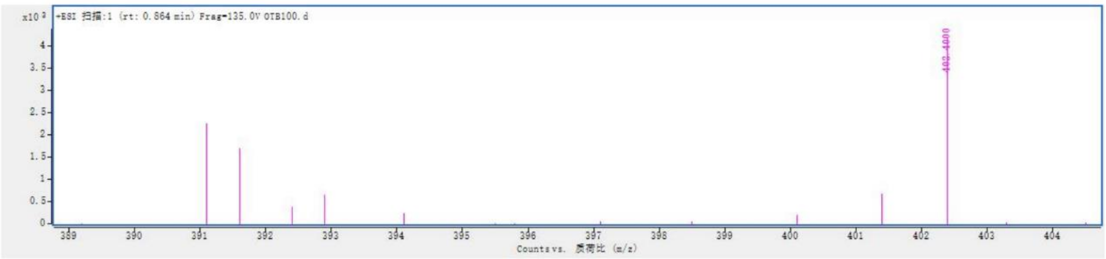

(h) 6-OH-OTB-quinone (from OTB)

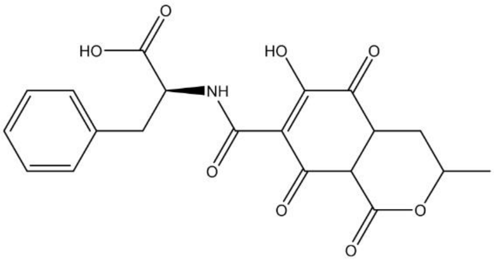

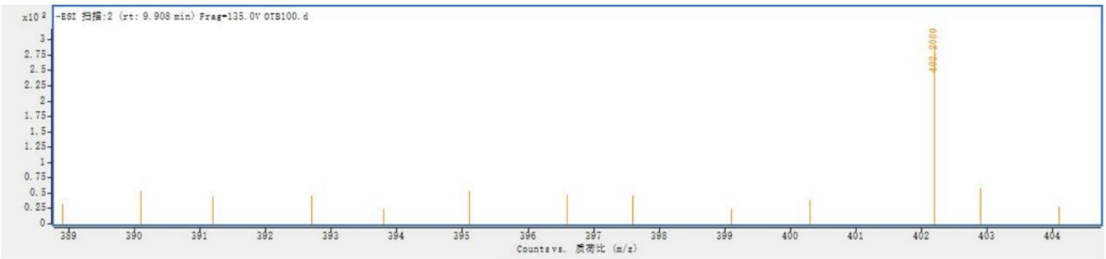

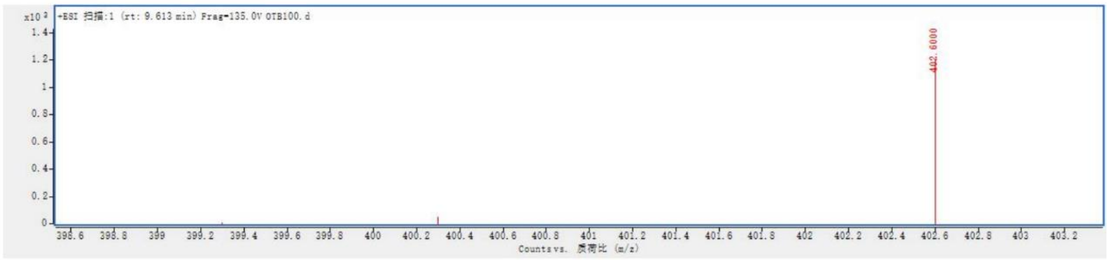

The MDA, SOD, ALT, AST, ALP and ROS were analyzed as follows:

MDA analysis: The principle is to use the reaction of MDA with thiobarbituric acid to generate a red-brown tri-methylpyridine compound for detection. The procedure used for analysis was as follows: Transfer the test cells to a pre-cooled centrifuge at 4°C, centrifuge at 2500 r/min for 10 min, and take the supernatant for testing. Dissolve the reagents according to the instructions of the MDA kit and balance at room temperature for 30 min. Incubate at 95°C for 40 min. After cooling, place in the centrifuge at 3500 r/min for 10 min and retain the supernatant. Calibrate and zero the spectrophotometer with distilled water, and measure the absorbance values of each tube at 532 nm. MDA content (nmol/ml) = (a1 - a2) / (b1 - b2) × c (10 nmol/ml) × N (a1: measured OD, a2: control OD, c: standard sample concentration, N: dilution factor of the sample before testing).

SOD analysis: The procedure used for analysis was as follows: Take the renal tubular epithelial cells (HK-2) of the test subject, centrifuge at 2500 rpm for 10 min, retain the supernatant for testing, and measure at 450 nm. SOD inhibition rate (%) = [(x1 - x2) - (y1 - y2)] / (x1 - x2) × 100% (x1: control, x2: blank control, y1: test, y2: blank test); SOD activity (U/mL) = SOD inhibition rate ÷ 50% × N1 (0.24 ml / 0.02 ml) × N2 (N1: dilution factor of the reaction system, N2: dilution factor before sample testing).

ALT, AST and ALP analysis: The procedure used for analysis was as follows: Thaw the calibrators and retain the supernatant. Centrifuge to collect the supernatant of the test human renal tubular epithelial cells (HK-2) from each treatment group. Then prepare the substrate buffer and cofactor/color developer in proportion and mix well to prepare the working reagent. The main wavelength of ALT and AST is 340 nm, the auxiliary wavelength is 405 nm, the delay time is 20-30 s, and the monitoring time is 2-5 min; for ALP, the main wavelength is 405 nm, the auxiliary wavelength is 505 nm, the reaction time is 1-3 min, and the temperature is 37°C. The activity of ALT, AST and ALP (U/L) = ΔA/min × V total / (ε × d × V sample) (ΔA: the increase in absorbance per minute or the consumption of substrate; d: the optical path (cm); for ALT and AST, ε: the molar absorptivity of NADH is 6.22×10^3 $L \cdot mol^{-1} \cdot cm^{-1}$; for ALP, ε: the molar absorptivity of p-nitrophenol is 18.8×10^3 $L \cdot mol^{-1} \cdot cm^{-1}$).

ROS of HK-2 cells analysis: The procedure used for analysis was as follows: Using a 10 μmol/L superoxide anion probe, add an appropriate volume of the diluted superoxide anion probe to the cell culture medium (fluorescence excitation spectrum = 300 nm, fluorescence emission spectrum = 610 nm). At the same time, add Hoechst staining for nuclei, with the final concentration of Hoechst33342 being 5 μg/ml (Ex = 350nm, Em = 461nm). Mix thoroughly. Place the cells in a 37°C incubator for 30 min of incubation. After staining, remove the staining solution, and wash the cells three times with serum-free medium to remove the superoxide anion probe that did not enter the cells. Capture images with an inverted fluorescence microscope for quantitative analysis.
